# Disrupted brain functional hub and causal connectivity in acute mild traumatic brain injury

**DOI:** 10.18632/aging.102484

**Published:** 2019-11-20

**Authors:** Fengfang Li, Liyan Lu, Huiyou Chen, Peng Wang, Yu-Chen Chen, Hong Zhang, Xindao Yin

**Affiliations:** 1 Department of Radiology, Nanjing First Hospital, Nanjing Medical University, Nanjing, China; 2 Department of Radiology, The Affiliated Jiangning Hospital of Nanjing Medical University, Nanjing, China

**Keywords:** mild traumatic brain injury, resting-state fMRI, degree centrality, Granger causality analysis, causal connectivity

## Abstract

There have been an increasing number of functional magnetic resonance imaging (fMRI) reports on brain abnormalities in mild traumatic brain injury (mTBI) at different phases. However, the neural bases and cognitive impairment after acute mTBI are unclear. This study aimed to identify brain functional hubs and connectivity abnormalities in acute mTBI patients and their correlations with deficits in cognitive performance. Within seven days after brain injury, mTBI patients (n=55) and age-, sex-, and educational -matched healthy controls (HCs) (n=41) underwent resting-state fMRI scans and cognitive assessments. We derived functional connectivity (FC) strength of the whole-brain network using degree centrality (DC) and performed Granger causality analysis (GCA) to analyze causal connectivity patterns in acute mTBI. Compared with HCs, acute mTBI patients had significantly decreased network centrality in the left middle frontal gyrus (MFG). Additionally, acute mTBI showed decreased inflows from the left MFG to bilateral middle temporal gyrus (MTG), left medial superior frontal gyrus (mSFG), and left anterior cingulate cortex (ACC). Correlation analyses revealed that changes in network centrality and causal connectivity were associated with deficits in cognitive performance in mTBI. Our findings may help to provide a new perspective for understanding the neuropathophysiological mechanism of acute cognitive impairment after mTBI.

## INTRODUCTION

Traumatic brain injury (TBI) is a traumatic brain structural injury or an injury in which other changes in brain function caused by external forces occur; TBI is a major reason for sustained disability and morbidity, both in military populations and civilians [1]. Mild TBI (mTBI) is the most common type of traumatic brain injury, representing 80%–90% of TBI cases [2]. Many mTBI patients may develop a wide range of cognitive deficits, such as deficits in executive function, attention, and working memory [3, 4]. Although most mTBI patients recover quickly, approximately 10%-15% of patients have disabling problems that persist for a long time [5]. However, to date, no obvious structural damage has been identified in mTBI patients when using traditional brain imaging techniques (i.e., computed tomography (CT) and magnetic resonance imaging (MRI)) [6]. Therefore, the cognitive problems of many mTBI patients may be underrated and thus have a substantial impact on the patients’ lives and social interactions. Nonetheless, the pathophysiologic mechanisms of mTBI that cause the disorder to be persistent remain poorly understood.

Previous neuroimaging studies using positron emission tomography (PET), magnetoencephalography (MEG), and functional magnetic resonance imaging (fMRI) have investigated the neuropathophysiological mechanisms implicated in subacute and chronic mTBI patients [7–9]. Resting-state fMRI (rs-fMRI), which reflects brain activity while an individual is resting and not performing a specific task, may reveal damage to cognitive function caused by mTBI [10]. Using rs-fMRI, multiple resting-state brain intrinsic networks relevant to the neural mechanisms of mTBI have been demonstrated, such as the default mode network (DMN) [11, 12], executive control network (ECN) [13], and motor network [14]. However, these results were variable due to the application of different approaches to rs-fMRI data analysis and the selection of pre- and post-processing procedures and heterogeneous samples. Mayer et al. implied that there was decreased functional connectivity (FC) within the DMN in chronic mTBI patients using seed-based rs-fMRI analysis [15]. Through independent component analysis (ICA), Palacios et al. found that mTBI patients in the subacute stage showed reduced connectivity in the ECN, the frontal nodes of the DMN and the parietal areas of the dorsal attentional network, as well as an increase in connectivity in the visual network [10]. Through the fractional amplitude of low-frequency fluctuations (fALFF), Madhavan et al. observed decreased fALFF in the motor, language, visual and salience networks for patients with higher symptom severity scores [16]. Heterogeneity among mTBI populations may lead to changes in network status. Based on these latest theories and observations, we speculate that the pathophysiological basis of acute mTBI may be due to disrupted brain function and network hubs.

The latest rs-fMRI studies identified abnormalities in the prefrontal cortex (PFC) associated with mTBI-related cognitive deficits, such as deficits in executive function, attention and working memory [17–20]. The PFC is a collection of interconnected new cortical regions that sends and receives projections from cortical motor systems, sensory systems, and many subcortical structures. The PFC helps to improve cognitive function and has been shown to be an important brain structure for performing executive functions [21]. Previous neuroimaging studies have confirmed the involvement of the PFC in the regulation and adaption of cognitive impairment after mTBI [22, 23]. Additionally considering the theory that the PFC is vulnerable to neural contusion, the PFC is considered a key region involved in mTBI. Therefore, exploring the role of the PFC in the brain functional network hubs of mTBI patients will provide valuable insights into the neural mechanisms of mTBI.

The ICA and seed-based FC approaches have been proved extremely useful in exploring FC patterns for specific components of interest. However, few studies have investigated the acute mTBI-related changes of whole-brain FC pattern or large-scale brain network. Degree centrality (DC) is a voxel-wise data-driven method for quantifying the importance of each node in a brain network [24]. This graph theory based on network analysis can evaluate the centrality of a network without selecting interested nodes or networks in advance. In addition, in order to examine the directional connectivity network involved in acute mTBI, Granger causality analysis (GCA) will be applied to evaluate the directional effects between simultaneously recorded time series. GCA allows the directional connections between different brain regions to be studied and the changes in brain networks to be dynamically observed, and GCA has been used to reveal causal relationships among brain regions in individuals with various diseases, such as autism, schizophrenia, Alzheimer’s disease (AD), and presbycusis [25–28]. However, it remains to be determined whether such DC and causal connection abnormalities can be observed at the early stages of mTBI.

Thus, in the present study, in order to unravel the details of the network hub and causal connectivity of brain function in mTBI patients in the acute stage, we used DC to quantify the importance of each node in the brain network in patients with acute mTBI, based on the strength of its connection to all the other voxels and then used GCA to analyze the causal connections and understand the directional aspects of these changes. We further aimed to determine whether DC and causal connection abnormalities are associated with cognitive dysfunction performance in acute mTBI patients. We hypothesized that (1) the intrinsic disconnection pattern of PFC plays a pivotal role in the brain functional network of mTBI patients at the acute stage. (2) DC and causal connection abnormalities would be associated with cognitive dysfunction performance in mTBI patients at the acute stage.

## RESULTS

### Demographics and neuropsychological characteristics

During this study, 95 patients with well-documented mTBI were identified. Among these patients, 70 patients were willing to participate in this study. Then, 6 patients were excluded because of excessive head motion artifacts, 5 patients were excluded because of a previous head injury, and 4 patients were excluded because of MRI contraindications. The remaining 55 mTBI patients obtained an MRI scan at an average of 3.47 days (range, 0–7 days) after head injury. A total of 41 healthy subjects matched by age, sex, and years of education were also assessed.

The basic demographic and cognitive performance characteristics of the mTBI group and HCs group are given in Table 1. There were no significant differences in age, sex or educational attainment between the mTBI group and the HCs group. Compared with the HCs, the mTBI patients at the acute stage performed worse on the MoCA scale (*p*=0.001). Among the subcategories of the MoCA test, our findings indicated that mTBI patients only showed significantly worse performance in visuospatial/executive (*p*=0.035) and attention (*p*=0.008) subcategories than did the HCs. There were no other significant differences in performance in naming, language, memory, abstraction or orientation between the mTBI group and HCs in this study.

**Table 1:** Demographic Characteristics and cognitive performance in patients with mTBI and HCs.

**Characteristic**	**mTBI (n=55)**	**Controls(n=41)**	***p*-value**
Age(years)	40.65±11.44	43.24±11.72	0.280
Education(years)	12.15±3.27	13.22±3.45	0.124
Sex(Female/ Male)	28/27	22/19	0.838
GCS Score	15	-	-
Time since injury(d)	3.47±1.89	-	-
MoCA scores	24.25±2.26	25.88±2.40	0.001^*^
Visuospatial/executive	3.69±0.84	4.10±1.02	0.035^*^
Naming	2.85±0.41	2.73±0.45	0.163
Attention	5.36±0.83	5.76±0.49	0.008^*^
Language	2.35±0.65	2.49±0.59	0.272
Abstraction	1.89±0.32	1.98±0.16	0.117
Memory	2.31±1.17	2.68±1.65	0.197
Orientation	5.80±0.40	5.90±0.30	0.175

Data are the mean ± standard deviation; *p<0.05. mTBI, mild traumatic brain injury; HCs, healthy controls; GCS, Glasgow Coma Scale; MoCA, Montreal Cognitive Assessment.

### Degree centrality analysis

**Figure 1:**
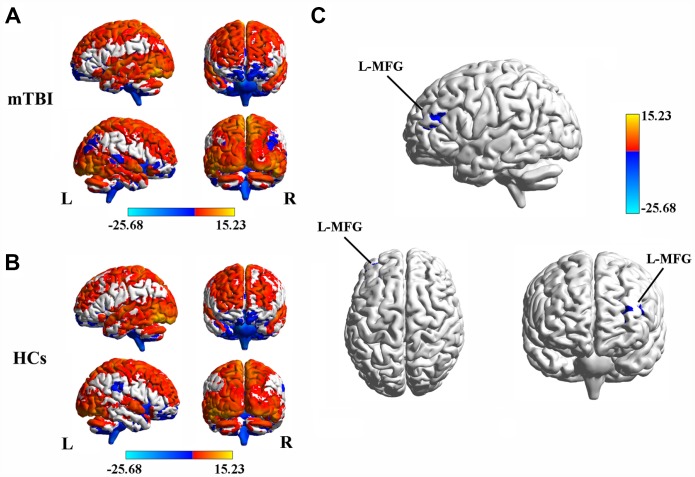
(**A**) Spatial distribution of the degree centrality (DC) in mTBI patients at the acute stage. (**B**) Spatial distribution of the DC in the healthy controls (HCs). (**C**) Significantly decreased DC within the left middle frontal gyrus (MFG) between acute mTBI patients and HCs. mTBI, mild traumatic brain injury; HCs, healthy controls.

In both mTBI patients (Figure 1A) and HCs (Figure 1B), the spatial distribution of the weighted DC was mostly localized in the PFC and temporal lobe areas. Specifically, after two-sample *t*-test analysis, significantly decreased DC within the left middle frontal gyrus (MFG) was found in mTBI patients at the acute stage compared to that of the HCs (Figure 1C and Table 2).

**Table 2:** Regions showing significant differences in degree centrality and causal connectivity between mTBI patients and HCs.

**Brain regions**	**BA**	**Peak MNI coordinates x, y, z (mm)**	**Peak T value**	**Voxels**
**Decreased DC**				
Left MFG	10	-33, 45, 18	-4.126	108
**Decreased FC (left MFG → whole brain)**				
Left MTG	20	-57, -12, -24	-3.4577	55
Left mSFG	10	-15, 63, 18	-4.0393	111
Left ACC	32	-6, 24, 30	-4.6747	113
Right MTG	39	54, -57, 3	-3.6263	157
**Increased FC (whole brain → left MFG)**				
Left ACC	32	-12, 24, 30	4.1316	81

A corrected threshold of *p*<0.05 was determined by Monte Carlo simulation. DC, degree centrality; FC, functional connectivity; BA, Brodmann’s area; MNI, Montreal Neurological Institute; MFG, middle frontal gyrus; IFG, inferior frontal gyrus; MTG, middle temporal gyrus; mSFG, medial superior frontal gyrus; ACC, anterior cingulate cortex.

### Causal connectivity analysis

**Figure 2:**
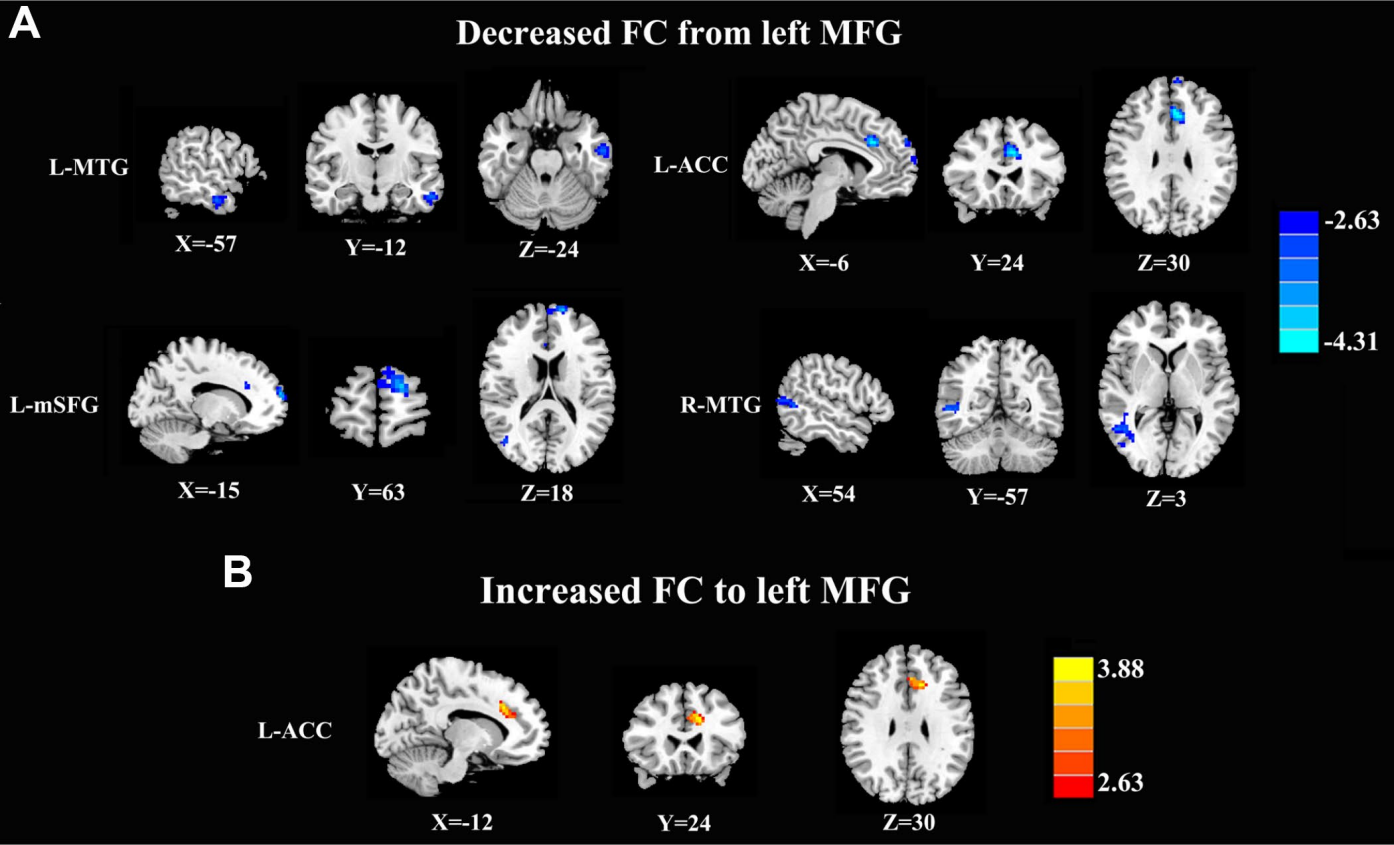
**Aberrant causal connectivity from the bilateral SFG in mTBI patients at the acute stage.** (**A**) Decreased causal connectivity from the left MFG to the L-MTG, R-MTG, L-ACC, and L-mSFG. (**B**) Increased causal connectivity from the left MFG to the left ACC. L, left; R, right; MFG, middle frontal gyrus; MTG, middle temporal gyrus; ACC, anterior cingulate cortex; mSFG, medial superior frontal gyrus.

The left MFG-to-whole brain GCA showed that patients with acute mTBI, compared with the HCs, demonstrated significantly decreased causal connectivity from the left MFG to the left middle temporal gyrus (MTG), right MTG, left medial superior frontal gyrus (mSFG), and left ACC. Next, whole brain-to-left MFG GCA showed that the acute mTBI group showed increased connectivity from the left ACC to the left MFG compared with the HC group, and we did not find a decreased feedback effect to the left MFG in the acute mTBI patients (Figure 2 and Table 2).

### Correlation results

**Figure 3:**
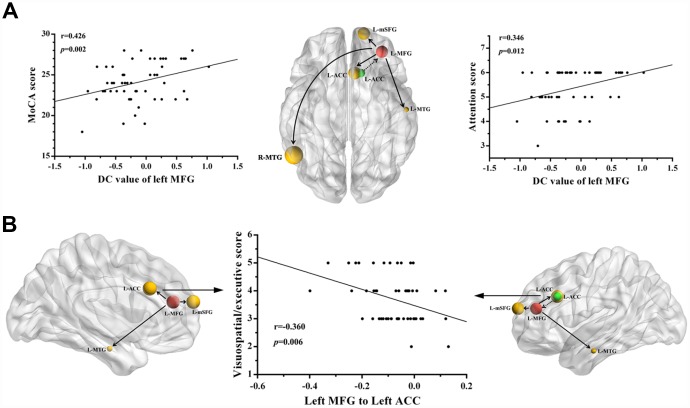
**Correlations between the changes in degree centrality (DC) and causal connectivity and deficits in cognitive performance in mTBI patients at the acute stage.** (**A**) The decreased DC in left MFG was positively correlated with the total MoCA scores (r = 0.426, *p* = 0.002) and the attention scores (r = 0.346, *p* = 0.012). (**B**) The visuospatial/executive score was negatively correlated with the decreased causal connectivity from the left MFG to left ACC (r = 0.360, *p* = 0.006). The full line represents the decreased causal connectivity and the dotted line represents the increased causal connectivity. L, left; R, right; MFG, middle frontal gyrus; MTG, middle temporal gyrus; ACC, anterior cingulate cortex; mSFG, medial superior frontal gyrus; MoCA, Montreal Cognitive Assessment.

The decreased DC of the left MFG was positively correlated with the total MoCA score (r=0.426, *p*=0.002) in patients with mTBI in the acute stage, and another positive correlation between deficits in attention (r=0.346, *p*=0.012) and the DC of the left MFG was also revealed (Figure 3A). In addition, the visuospatial/executive score was negatively correlated with decreases in causal connectivity from the left MFG to the left ACC (r=-0.360, *p*=0.006) (Figure 3B). The other regions with abnormal causal connectivity revealed no significant correlations with the MoCA scores. However, no significance was observed after Bonferroni multiple comparisons correction due to the relatively strict calculation.

## DISCUSSION

This is the first study to use both DC and GCA approaches to explore intrinsic brain functional hubs and causal connectivity in mTBI patients at the acute stage. Using DC analysis, we found significantly decreased network centrality within the left MFG region in acute mTBI patients than in HCs. Using the GCA algorithm, we employed the left MFG as a seed to examine its causal effect with the whole brain. Specifically, the left MFG showed decreased causal connectivity with several regions, including the prefrontal, temporal and cingulate cortex. In addition, the left ACC showed significantly increased causal connectivity to the left MFG. Of note, the DC abnormalities in the left MFG and the disrupted causal connectivity between left ACC and left MFG were significantly correlated with cognitive function performance in mTBI patients at the acute stage. The current study could provide deeper insights into this issue and further confirm the concept that the acute mTBI is a pathophysiological process related to aberrant brain connectivity.

DC is the most direct measure to characterize node centrality in a network analysis. The higher the degree centrality a network node has, the more important the node is in the network. Numerous studies over the past century have confirmed the idea that the human brain network consists of neurons connected in complex patterns that support different cognition functions [29]. Our findings showed significantly decreased DC in the left MFG region of mTBI patients than in that of HCs, suggesting that the left MFG is the main brain functional hub affected by acute mTBI, which is consistent with the hypothesis that the dysconnectivity pattern of the PFC is involved in acute mTBI. However, only unilateral DC abnormalities were found in our study. A possible reason for this result is that the locations of the forces that caused head trauma in the patients included in this study were not completely consistent and need to be further explored. PFC is considered a key area by many TBI studies, which shows that the PFC is associated with many aspects of posttraumatic symptoms and cognitive impairment in individuals with mTBI [30, 22]. Harm et al. demonstrated significantly lower activation levels within the medial PFC in patients with mTBI in the subacute stage postinjury compared to HCs by using GLM analysis [17]. Moreover, Sullivan and colleagues found that chronic blast-related mTBI patients showed higher deactivation levels within the left dorsomedial PFC [13].

In the current study, we observed that decreased DC in the left MFG region was positively associated with the total MoCA score and attention score among patients with mTBI in the acute stage. These findings were consistent with the role of the PFC in the development of persistent cognitive and emotional impairment after a concussion. Consistent with this notion, previous studies have also reported functional alterations within the PFC region, both in moderate/severe TBI and in mild TBI, and such changes have been linked to cognitive performance [31–33]. Moreover, there is also evidence of structural and neurochemical abnormalities in the PFG and a relationship between the structural and neurochemical changes and cognitive performance [21]. Dall et al. demonstrated increased thickness within the PFC regions and weak correlations between the amount of PFC thickening and cognitive performance in chronic mTBI patients [31]. Popesccu et al. indicated that mTBI patients showed reduced alpha-band power in the bilateral MFG by using resting-state MEG, and the alpha-band power in the MFG was negatively correlated with scores reflecting symptoms of emotional numbing [34]. Therefore, all related studies have suggested that the PFC, specifically the MFG, might be an important part of cognitive and emotional regulation circuits; changes in this region may be associated with characteristic symptoms of cognitive impairment. Meanwhile, consistent with these valuable findings, our results are instructive for understanding the specific role of MFG abnormalities in patients with acute mTBI and suggest that MFC may provide valuable insights into the neural mechanism of acute mTBI.

In the current study, we used the GCA method to study the directed functional connection of the brain network in mTBI patients at the acute stage. The left MFG-to-whole brain GCA showed that left MFG activity predicted subsequent decreases in activity in the frontal cortex (ACC and mSFG) and temporal cortex (MTG). Previous neuroimaging studies have found that abnormalities in the frontal cortex may be the cause of cognitive impairment after mTBI. First, it has been revealed that the ACC regulates activity in the executive areas of the brain that direct attention and produce responses [35]. Changes in the ACC have been found in the MEG signals between combat-related mTBI patients and HCs by using MEG and N-back working memory tasks [36]. Furthermore, we found a negative correlation between the visuospatial/executive score and decreases in connectivity from the MFG to the ACC, which also emphasized the pivotal role of the ACC in acute mTBI. However, the exact mechanism for this correlation result is unclear. We speculated that this negative correlation may also be a compensatory effect during the first few days following mTBI drawing on the previously study [37], but this compensatory mechanism needs to be confirmed in future studies. Our current results also indicated that acute mTBI patients showed decreased causal connectivity from the left MFG to the left mSFG. This finding was in line with that of another study with rs-fMRI, which showed significantly decreased ALFFs in the SFG of mTBI patients compared with those in the SFG of HCs [38]. Popescu and colleagues’ analysis of MEG data showed that the power of the alpha band in the SFG was generally lower in mTBI patients than in HCs, and the changes in alpha-band power were negatively associated with scores reflecting symptoms of emotional numbing [34]. All these studies supported the idea that the left MFG is an important part of information processing and can coordinate working memory (e.g., short-term memory) and other sources of information that are particularly important for attention and emotional maintenance [39].

Previous fMRI studies have suggested that MTG located in the posterior temporal lobe plays a vital role in auditory and motor processing functions [40]. However, recent neuropsychological and imaging literature suggested that MTG may be important for various aspects of working memory, visual and emotional cognition perception [41]. In the present study, we also found that the mTBI group showed reduced causal connectivity from the left MFG to both sides of the MTG. One previous study provided evidence that the mTBI group had significantly higher levels of fractional anisotropy and axial diffusivity in the MTG white matter region, and the diffusion alterations were correlated with acute symptom burden in mTBI patients [42]. By using brain SPECT imaging, Bonne and colleagues found that mTBI patients in the chronic stage had reduced relative blood perfusion in the MTG [43]. All these studies suggested that the structural and functional abnormalities in the MTG may be one of the underlying mechanisms of cognitive function impairment, and given the interactions between multiple functional networks that are known to exist in the brain, this change seems reasonable. Therefore, we speculated that the MTG may be a modal semantic center that binds information from different sensory modes together. Nevertheless, the association of the MTG with mTBI has not been substantially elucidated, and further research is needed to clarify the role of the MTG in mTBI.

Additionally, the whole brain-to-left MFG GCA also revealed that acute mTBI patients had significantly increased causal connectivity from the left ACC to the left MFG, suggesting that activity in the left ACC may predict subsequent increases in activity in the left MFG. Hyper-connectivity in TBI patients has previously been reported in task-related brain function and resting-state brain function. Chen et al. found that mTBI patients showed increased activity in several areas of the same region, including the pre-exercise area and the motor cortex, assuming that the activity in these areas represented a compensation mechanism because TBI patients performed as well on memory tasks as healthy controls [37]. Sullivan et al. showed enhanced negative coupling FC between the dorsomedial PFC and the dorsal ACC in blast-related mTBI patients, and the authors postulated that the enhanced negative coupling in mTBI patients serves as a compensatory mechanism for successful task performance [13]. In a recent study of cognitive control in civilians with mTBI, the enhanced activation in the mTBI group was also interpreted to reflect a compensatory mechanism, but this activation was observed in the parietal subcortex, which is not part of the functional network discussed in the current study [44]. The use of compensatory mechanisms that substitute for cognitive resources is also thought to explain functional imaging results in studies in moderate to severe TBI patients [45]. Consistent with the above notion, our results provide further evidence that compensatory activity may allow normal levels of some cognitive function performance to be maintained in acute mTBI patients.

There are several limitations in our study. First, our experimental design is cross-sectional and limited in sample size, so it is difficult to make direct causal inferences about the relationship between brain functional network structures and cognitive impairment characteristics in patients with mTBI. Additional longitudinal studies involving more subjects are required in the future. Second, our present study is limited to patients with mTBI in different populations, who showed different injury mechanisms and different brain injury sites. Heterogeneity of the injury mechanisms may minimize the generalization of observed results to any particular injury mechanism. Distinguishing the damage models and mechanisms of mTBI and maintaining homogeneity in the study population will allow better inferences to be made for different types of injuries. Third, the meaning of causal connectivity by GCA in the static state is not completely clear, and there are still some deficiencies. It is unclear whether low-pass filtering is the best method for resting fMRI data of GCA, and further studies are needed to clarify this meaningful view. Functional magnetic resonance imaging and electrophysiology require further research to elucidate the link between causal connections and neuronal activity. Finally, cognitive impairment after mTBI is very subtle, and our MoCA cognitive assessment method may not be sufficiently sensitive to identify these differences comprehensively. Therefore, in the future, it will be worthwhile to conduct additional studies using neuropsychological assessments and more sensitive cognitive performance indicators.

## CONCLUSIONS

To summarize, we have observed crucial functional network hub and causal connectivity aberrations within several regions revealed by DC and GCA in patients with mTBI at the acute stage. Compared to HCs, acute mTBI patients showed decreased FC strength in the left MFG region and abnormal directionality of influence both from and to the left MFG. Furthermore, decreased DC values in the MFG and altered causal connectivity from the left MFG to the left ACC were correlated with deficits in cognitive performance. The abnormalities in brain functional network structures are helpful in better understanding the neuropathophysiological mechanism of cognitive impairment after mTBI at the acute stage, which may help diagnose and evaluate the degree of cognitive impairment and recovery of patients to a certain extent.

## MATERIALS AND METHODS

### Subjects and clinical data

The current study was approved by the Institutional Review Board of Nanjing Medical University. Written informed consent was obtained from each subject before their participation in the study protocol.

We recruited 55 patients with mTBI (27 males and 28 females, 40.65 ± 11.44 years) at the acute stage from the emergency department of our hospital between January 2018 and May 2019. The inclusion criteria for patients were as follows: (1) age of 20 or older; (2) right-handedness before injury; (3) the presence of impact to the head; (4) an initial Glasgow Coma Score [GCS] of 13–15 in the emergency department; and (5) mTBI that was evaluated initially in the emergency room (posttraumatic amnesia <24 h, loss of consciousness<30 minutes, or recorded alteration of mental status (i.e., confused, dazed, or disoriented)). In addition, all patients were required to have a CT scan as part of their clinical evaluation. The exclusion criteria were as follows: (1) a documented history of a previous brain injury, the use of neuropsychological or psychoactive medications, a neurological disorder, or concurrent substance abuse; (2) history of drug or alcohol abuse; (3) history of the use of a sedative before a hospital or emergency department visit; and (4) MRI contraindications. Additionally, 41 age-, sex-, and educational attainment-matched healthy subjects (all right-handed) (19 males and 22 females; 43.24 ± 11.72 years) were recruited as controls. The control participants met the same exclusion criteria applied to the patient group. All control subjects underwent the same neuroimaging protocol as the patients. Demographic characteristics and outcome measurements of the mTBI and healthy control groups were summarized in Table 1.

### Cognitive function assessment

Given the emergency care setting, a complete neuropsychological assessment was not feasible. Therefore, we used a short instrument called the Montreal Cognitive Assessment (MoCA) to assess all mTBI patients’ neurocognitive status. The MoCA was developed by Nasreddine et al. based on clinical experience and Mini-Mental State Examination cognitive ratings and items [46]. It is a brief yet comprehensive cognitive instrument used to assess the level of impairment in neurological populations. The test lasts approximately 10 minutes and has a maximum score of 30 points. A score greater than or equal to 26 indicates a cognitively normal state, and a lower score indicates poorer cognitive performance [46]. The MoCA evaluates several categories of functionality, including visuospatial/execution, language, attention, naming, abstraction, memory (short-term immediate and delayed recall), and location of time and place [47]. All subjects underwent the same cognitive function assessment within seven days after mTBI.

### MRI acquisition

MRI data were acquired using a 3.0 T MRI scanner (Ingenia, Philips Medical Systems, Netherlands) at our hospital. The type of the receiver coil was digital head coil 3.0T and the parallel imaging was employed. Scanner noise and head motion were reduced using earplugs and foam padding. All subjects were asked to close their eyes, lie quietly without falling asleep, not think about anything in particular, and avoid any head movements during the scan. Structural images in this study were acquired using a three-dimensional turbo fast echo (3D-TFE) T1 WI sequence with high resolution parameters as follows: repetition time (TR) = 8.1 ms; echo time (TE) = 3.7 ms; thickness = 1 mm; slices = 170; gap = 0 mm; flip angle (FA)= 8°; FOV = 256 mm × 256 mm; and acquisition matrix = 256 mm × 256 mm. The structural sequence scan took 5 min and 28 s. Axial functional images were obtained using gradient echo-planer imaging (EPI) sequences, and the scanning parameters were as follows: slices = 36; thickness = 4 mm; TR/TE = 2000/30 ms; gap = 0 mm; acquisition matrix = 64 × 64; field of view (FOV) = 240 mm × 240 mm; and FA = 90°. The fMRI sequence scan took 8 min and 6 s.

### Functional data preprocessing

Functional data analyses were conducted with the Data Processing & Analysis for Resting-State Brain Imaging (DPABI_V2.3_170105) [48] and statistical parametric mapping (SPM8, http://www.fil.ion.ucl.ac.uk/spm) packages. A total of 240 volumes were scanned, and the first 10 volumes were discarded to balance the initial MR signal and subject's adaptation to the scanner. Afterwards, the remaining 230 consecutive volumes were used for data analysis. Then, the following procedures were performed: slice-timing adjustment, head motion correction adjustment, Montreal Neurological Institute (MNI) template space normalization (resampling voxel size = 3 × 3 × 3 mm^3^), isotropic Gaussian kernel smoothing (full width at halt maximum (FWHM)= 6 mm), and detrending and filtering (0.01-0.08 Hz). Subjects with head motion > 2.0° of rotation or 2.0 mm of translation in any direction were excluded. Finally, several sources of spurious variances were removed by linear regression, which included six head motion parameters, and average signals from white matter, cerebrospinal fluid, and whole brain.

### Degree centrality analysis

We limited voxel-wise centrality analyses to a predefined gray matter (GM) mask that included tissues with a GM probability >20%, as previously described [24]. In the mask, a separate network centrality map was generated in voxel-wise mode. First, whole brain correlation analysis based on the voxels was carried out for functional operation after preprocessing. The time course of each individual voxel of each participant was correlated with the time course of another individual voxel, resulting in a correlation matrix. Then, the undirected adjacency matrix was obtained by thresholding each association at r > 0.25 [49]. We chose a high threshold to prevent voxels with low temporal correlations due to signal noise from being counted. Then, we calculated the binary DC value of the whole brain network. A graph of the DC values for each gray matter voxel of each participant was obtained. Finally, these voxel-wise DC maps were converted into a z-score map for group comparisons. As previously reported, negative correlations were not included in DC calculations because their interpretation is not clear and has an adverse effect on the reliability of test-retest [50].

First, we estimated the spatial distribution of the mean DC in the mTBI group and the healthy group, respectively. The z-value of each individual was entered into the SPM8 software, and a random effects one-sample t-test was performed for each voxel to generate an average DC map for each group. The multiple comparison corrections were performed using false discovery rate (FDR) criterion and set at a threshold of *p*<0.01. In addition, to find the damaged brain center region, a two-tailed two-sample t-test was performed to study the differences in DC maps between mTBI patients and healthy controls (HCs). Between-group comparisons of the DC maps were performed in SPM8 software using general linear model (GLM) analysis, and age, sex, and educational attainment were included as unwanted covariates. Multiple comparison corrections were performed using the AlphaSim program determined by Monte Carlo simulation. Statistical maps of the two-sample t-test were created using a combined threshold of *p*<0.01 and a minimum cluster size of 40 voxels, yielding a corrected threshold of *p*<0.05. For between-group analyses, a mask was created by combining the significant clusters in the two groups, which were obtained from the one-sample t-test results.

### Causal connectivity analysis

To further investigate the effects of FC directionality, we applied GCA to assess alterations in causal connectivity. Based on the results of the DC analysis, we selected a seed region that showed a significant difference between mTBI patients and HCs (left MFG: MNI coordinates (*x, y, z*) -33, 45, 18). The causal connectivity was analyzed by using REST-GCA in the REST toolbox [51]. In this study, the time series of the left MFG was defined as the seed time series x, and the time series y represented the time series of all voxels in the brain. The linear direct effect of x on y (F_x→y_) and the linear direct effect of y on x (F_y→x_) were voxels calculated by voxels in the brain. Therefore, two Granger causality maps were generated based on the impact metric for each participant. The residual-based F was normalized (F’) and normalized to the Z score for each voxel (Z_y→x_ and Z_x→y_), subtracting the global mean F’ values, divided by standard deviation.

For group analyses of causal connectivity, the mean values of the Z_y→x_ and Z_x→y_ maps were calculated for each group. All four Granger causality maps, including two for each direction and two for each group, were obtained (the left MFG included both Z _y→x_ and Z _x→y_ for both the mTBI patients and HCs). These Granger causality maps were then entered into the SPM8 software for group comparisons. A random effects two-sample t-test was performed for each voxel to determine the difference in the causal connectivity of MFG between mTBI patients and HCs, with sex, age, and educational attainment included as unwanted covariates. Multiple comparison corrections were performed by Monte Carlo simulation using the AlphaSim program, with a corrected threshold of *p* < 0.01 and a minimum cluster size of 40 voxels, yielding a corrected threshold of *p*<0.05.

### Correlation analysis

To explore the relationship between clinical cognitive deficit characteristics of mTBI patients and DC and causal connectivity measures, the areas showing significant differences in causal connections or DC between groups were extracted. We calculated the correlation between the mean z-values within these clusters and the cognitive deficit characteristics of each mTBI patient using Pearson correlation analysis in SPSS software (version 18.0; SPSS, Chicago, IL, USA). *P* < 0.05 was considered statistically significant and was corrected for age, sex, and educational attainment. Bonferroni correction for multiple comparisons was applied to the correlation analysis.
